# Machine learning based prediction of antimicrobial resistance *in Klebsiella spp*.: a five-year retrospective study

**DOI:** 10.3389/fpubh.2026.1865551

**Published:** 2026-07-01

**Authors:** Munerah M. Alfadhel, Mohammed F. Aldawsari, Ehssan Moglad

**Affiliations:** Department of Pharmaceutics, College of Pharmacy, Prince Sattam Bin Abdulaziz University, Al-Kharj, Saudi Arabia

**Keywords:** antimicrobial resistance (AMR), Extreme Gradient Boosting (Xgboost), *Klebsiella*, Random Forest, Saudi Arabia, Support Vector Machine (SVM)

## Abstract

**Introduction:**

*Klebsiella* species are well-recognized pathogens implicated in both healthcare-associated and community-acquired infections, and they contribute substantially to the global burden of antimicrobial resistance. In this study, we investigated the epidemiology, temporal resistance trends, multidrug resistance profiles, and the application of machine learning (ML) approaches to predict antimicrobial susceptibility among Klebsiella isolates in Al-Kharj, Saudi Arabia.

**Methods:**

A retrospective analysis conducted using routine microbiology laboratory data collected between 2019 and 2024. Antimicrobial susceptibility testing included multiple agents across major antimicrobial classes, allowing classification of isolates into defined resistance phenotypes. Multidrug-resistant (MDR), extensively drug-resistant (XDR), and pan-drug-resistant (PDR) profiles were determined using standard class-based definitions. In parallel, several supervised machine learning models were developed and evaluated, including Random Forest, Support Vector Machine (SVM), Gradient Boosting, Extreme Gradient Boosting (XGBoost), Light Gradient Boosting Machine (LightGBM), Extra Trees Classifier, Classifier Chains (multilabel classification), Deep Neural Network (DNN), Voting Ensemble, and Convolutional Neural Network (CNN). Models were trained using the training dataset and subsequently evaluated on the testing dataset to assess predictive performance and generalizability.

**Results:**

Over the five-year study period, *Klebsiella* species accounted for 2,646 isolates (13.7%) of all clinical isolates, with *Klebsiella pneumoniae* representing the predominant species. Urinary tract infections were the most frequent source (45.5%), followed by blood cultures (15.4%), respiratory samples (14%), and wound and soft tissue specimens (12.2%). High resistance rates were observed for ceftazidime, whereas most other antibiotics demonstrated moderate resistance levels. Tigecycline, and Ceftriaxone showed the lowest resistance rates. Among *Klebsiella* spp. isolates, 57.8% were classified as multidrug-resistant, 1.9% as extensively drug-resistant, and no pan-drug-resistant isolates were identified. Among the evaluated models, the best overall model was XGBoost with an accuracy of 0.70, precision of 0.65, recall of 0.64, an F1-score of 0.64, and a ROC-AUC of 0.74.

**Conclusions:**

Collectively, these findings underscore the increasing clinical burden of *Klebsiella* infections and demonstrate the potential of boosting-based machine learning algorithms, particularly XGBoost and LightGBM, for accurate prediction of antibiotic resistance. Integration of these models into clinical decision-support systems and antimicrobial stewardship programs may facilitate timely and appropriate antimicrobial therapy, thereby improving patient outcomes and promoting more rational antibiotic use.

## 1 Introduction

*Klebsiella spp*. are Gram-negative, encapsulated member of the *Enterobacteriaceae* family, and is a common colonizer of the human gastrointestinal tract. Despite its commensal presence, *Klebsiella* species are important opportunistic pathogens capable of causing severe infections, including pneumonia, bloodstream infections, urinary tract infections, and sepsis, particularly among hospitalized and immunocompromised patients. Over the past two decades, *K. pneumoniae* has emerged as a major global public health concern due to its exceptional capacity to acquire and disseminate antimicrobial resistance determinants (1, 2).

The global burden of antimicrobial-resistant *Klebsiella* infections is substantial. The 2019 Global Burden of Disease study estimated that antimicrobial resistance was directly responsible for 1.27 million deaths worldwide, with *K. pneumoniae* ranked among the leading contributors to resistance-associated mortality (3). The World Health Organization (WHO) has consequently classified carbapenem-resistant and extended-spectrum β-lactamase (ESBL)-producing K. pneumoniae as critical-priority pathogens, reflecting the limited therapeutic options and high mortality associated with these infections (4, 5).

Multidrug-resistant (MDR) *Klebsiella* strains frequently harbor extended-spectrum β-lactamases (ESBLs) and carbapenemases, including KPC, NDM, and OXA-48-like enzymes, alongside additional resistance mechanisms affecting fluoroquinolones, aminoglycosides, and trimethoprim–sulfamethoxazole (1, 2). These determinants often co-localize on mobile genetic elements, promoting rapid dissemination within and between healthcare facilities. Consequently, infections caused by MDR *Klebsiella* are associated with prolonged hospitalization, increased healthcare costs, and markedly elevated mortality rates (6).

In the Middle East, particularly in Saudi Arabia, *Klebsiella* species have emerged as prominent causes of healthcare-associated infections. Several studies from tertiary care settings have reported high rates of multidrug resistance and carbapenem resistance (7–9). National surveillance and hospital-based investigations further indicate a persistent rise in resistance to β-lactams, fluoroquinolones, and aminoglycosides, while carbapenem-resistant K. pneumoniae has become a significant therapeutic challenge (8, 10). Taken together, these findings underscore the urgent need for robust epidemiological surveillance and advanced analytical approaches to strengthen antimicrobial stewardship and infection control strategies.

Recent advances in machine learning (ML) have provided powerful tools for analyzing large-scale antimicrobial susceptibility datasets. ML-based models have shown considerable promise in predicting resistance phenotypes, uncovering complex resistance patterns, and supporting real-time clinical decision-making (11–13). Recent studies have explored the application of machine learning for antimicrobial resistance prediction. For example, CART and Random Forest models have been used to predict extended-spectrum β-lactamase (ESBL)-producing and multidrug-resistant bacterial infections based on microbiological and clinical characteristics, demonstrating promising predictive performance. However, most previous studies have focused on specific resistance phenotypes, such as ESBL production, whereas data on machine learning-based prediction of antimicrobial resistance patterns in *Klebsiella* spp. remain limited, particularly in regional healthcare settings (14). Integrating longitudinal surveillance data with ML-driven predictive frameworks may facilitate earlier detection of emerging resistance trends and improve empirical antibiotic selection, particularly for high-risk pathogens such as *Klebsiella* species. Accordingly, this study aims to characterize local resistance patterns, evaluate temporal trends, and develop predictive models to support effective antibiotic therapy and antimicrobial stewardship interventions.

## 2 Methods

### 2.1 Study design and data collection

**Figure 1:**
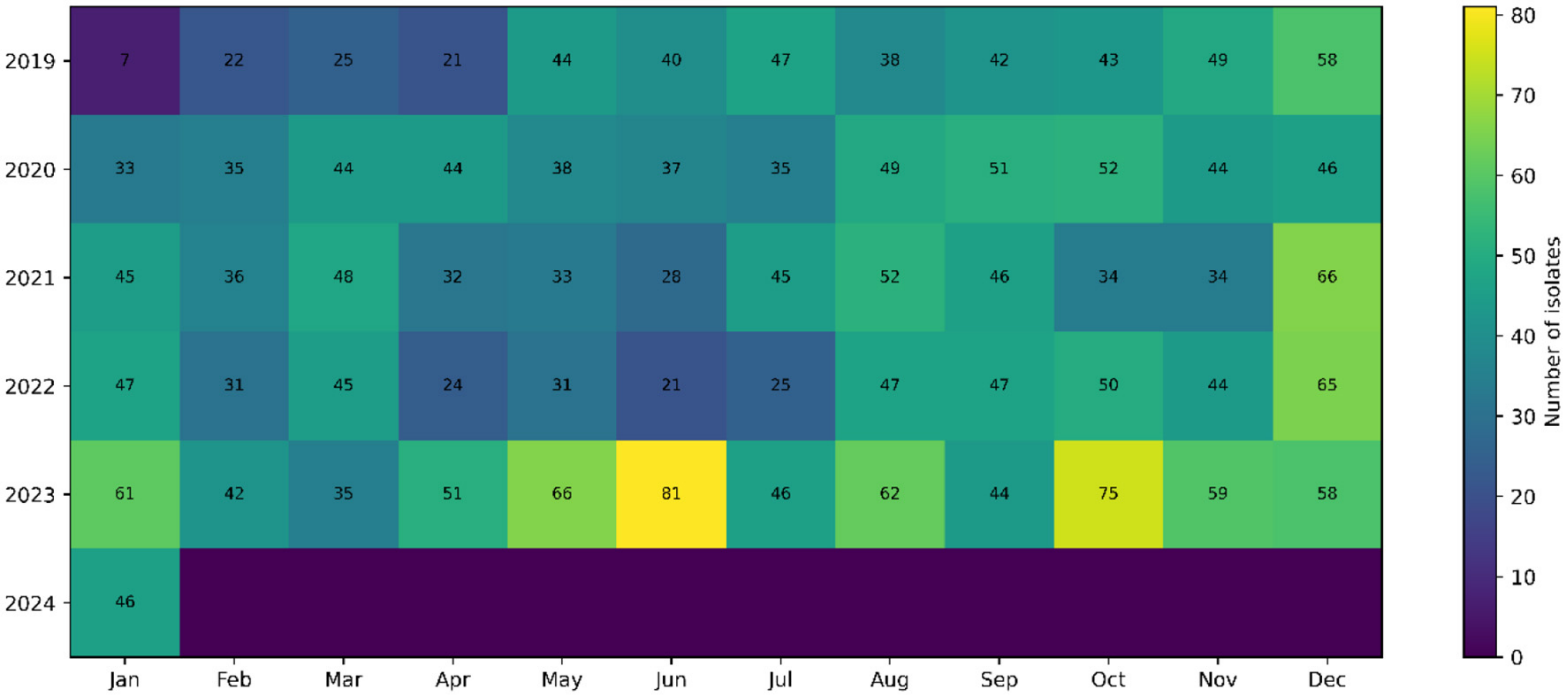
Heatmap illustrating the monthly distribution of *Klebsiella* spp. isolates collected between 2019 and 2024. Color intensity represents the number of isolates per month, with non-zero values annotated.

A five-year retrospective study was conducted between January 2019 and January 2024 using data retrieved from the clinical microbiology laboratory databases of three major tertiary hospitals in Al-Kharj, Saudi Arabia. Of the 18,003 antimicrobial susceptibility testing (AST) records reviewed for all isolated Gram-positive and Gram-negative bacteria, 3,204 *Klebsiella spp*. isolates. To avoid overrepresentation of repeated isolates, duplicate records were excluded when they shared the same collection date, patient gender, specimen source, and identical antimicrobial susceptibility profiles. Following deduplication, 2,646 unique isolates were retained from the initial dataset (Figure 1). These isolates were obtained from a range of clinical specimens collected from both male and female patients at various time points throughout the study period (13).

### 2.2 Antibiotics susceptibility test

Antimicrobial susceptibility testing for Klebsiella isolates was performed as part of routine clinical diagnostic procedures in the participating hospitals. AST was conducted using standardized laboratory methods in accordance with the clinical and laboratory standards institute (CLSI) guidelines. Results were interpreted using CLSI clinical breakpoints, and isolates were classified as susceptible (S), intermediate (I), or resistant (R). (15).

The antimicrobial panel included agents commonly used in the treatment of Gram-negative infections and spanned multiple antimicrobial classes, including Antimicrobial susceptibility testing was performed against a comprehensive panel of antibiotics routinely used in clinical practice. The tested agents were 24 different antibiotics included amikacin (AMK), amoxicillin–clavulanate (AMC), ampicillin (AMP), cefazolin (CZO), cefotaxime (CTX), aztreonam (AZT), ceftriaxone (CZX), cefoxitin (FOX), cefuroxime (CXM), ciprofloxacin (CIP), colistin (COL), ertapenem (ERT), gentamicin (GEN), ceftazidime (CAZ), levofloxacin (LVX), meropenem (MEM), imipenem (IPM), nitrofurantoin (NIT), norfloxacin (NOR), piperacillin–tazobactam (TZP), tobramycin (TOB), tetracycline (TET), tigecycline (TGC), and trimethoprim-sulfamethoxazole (SXT). Isolates with missing AST results for specific agents were retained in the analysis, and resistance classifications were determined based on available tested agents (Table S1).

### 2.3 Definitions of resistance phenotypes

Multidrug-resistant (MDR), extensively drug-resistant (XDR), and pan-drug-resistant (PDR) phenotypes were defined according to internationally accepted class-based criteria. MDR was defined as resistance to at least one agent in three or more antimicrobial classes, whereas XDR referred to resistance to all but one or two classes. PDR was defined as resistance to all tested antimicrobial classes (16). Carbapenem resistance was defined as resistance to at least one carbapenem agent, such as imipenem, meropenem, or ertapenem, when tested (17).

### 2.4 Statistical analysis

Descriptive statistics were applied to summarize isolate characteristics, specimen sources, species distribution, and resistance patterns. Categorical variables were presented as frequencies and percentages, and temporal trends were assessed according to the year of isolation. The prevalence of multidrug resistance (MDR) was also compared across species groups and specimen types. Heatmaps and trend plots were generated to visualize resistance distributions and temporal patterns. Statistical analyses were performed using Python-based analytical tools, and a two-sided *p*-value < 0.05 was considered statistically significant where applicable.

### 2.5 Machine learning analysis

#### 2.5.1 Dataset description

The dataset comprised 2,646 clinical samples, each containing information on organism type and antibiotic susceptibility results. Initially, 24 antibiotics were considered; however, due to substantial missing data, 15 antibiotics with the lowest proportion of missing values ( ≤ 26%) were selected for subsequent modeling (Table S2, Figure S1).

#### 2.5.2 Data preprocessing

Before model development, the dataset underwent a structured preprocessing pipeline to ensure data quality and analytical consistency. Missing antimicrobial susceptibility results were not imputed and were excluded from model training and evaluation. Predictor variables with excessive missingness were removed during feature selection, and only variables with acceptable completeness were retained for model development.

For machine learning model development, antimicrobial susceptibility results were converted into a binary outcome. Isolates classified as resistant (R) and intermediate (I) were grouped into a single non-susceptible category, while susceptible (S) isolates formed the second category. This approach was adopted to reduce class imbalance and facilitate robust binary classification, particularly for antibiotics with a limited number of intermediate isolates.

#### 2.5.3 Train-test split

The dataset was divided into training and testing subsets using multilabel stratified sampling to preserve the distribution of resistance patterns across all antibiotics. This strategy ensured a balanced representation of labels in both subsets and enhanced model generalization. The dataset was partitioned as follows: Training set: 80%

Testing set: 20%

The training set was used to train the models, while the testing set was used for final performance evaluation on unseen data.

#### 2.5.4 Model development

Multiple machine learning and deep learning models was developed to predict antibiotic resistance patterns. These algorithms were selected to capture both linear and non-linear relationships within the dataset and to enable performance comparisons across diverse modeling approaches.

Ten models were implemented and evaluated: Random Forest, Gradient Boosting, Extreme Gradient Boosting (XGBoost), Support Vector Machine (SVM), Light Gradient Boosting Machine (LightGBM), Extra Trees Classifier, Classifier Chains (multilabel classification), Voting Ensemble, Deep Neural Network (DNN), and Convolutional Neural Network (CNN). Each model was trained using the training dataset and subsequently evaluated on the testing dataset to assess predictive accuracy and generalization performance (13, 18–21). To handle class imbalance, oversampling techniques were applied to the training data.

#### 2.5.5 Model input variable

The predictor variables included source of sample, month, year, gender, organism name, and quarter, which served as inputs for predicting antibiotic resistance outcomes. Their relative importance was subsequently assessed using aggregated feature importance scores derived from tree-based models (Table S3).

#### 2.5.6 Evaluation metrics

For model evaluation, isolates lacking laboratory-confirmed susceptibility results for a given antibiotic were considered to have unavailable labels and were excluded from performance calculations using masking procedures. Consequently, accuracy, precision, recall, F1-score, ROC-AUC, and confusion matrix analyses were calculated only for isolates with available reference susceptibility results.

Model performance was evaluated using multiple metrics to provide a comprehensive assessment of predictive accuracy and classification performance. The F1-score was designated as the primary evaluation metric because it balances precision and recall, making it particularly appropriate for imbalanced datasets. Additional performance measures, including accuracy, precision, recall, ROC-AUC, and confusion matrices, were used to further characterize model discrimination and classification performance.

The following metrics were used for evaluation:

F1 Score (Primary metric): measures the balance between precision and recall, offering a robust indicator for imbalanced classification tasks.

Receiver Operating Characteristic – Area Under the Curve (ROC-AUC): assesses the model's ability to distinguish between resistant and sensitive classes across varying threshold values.

Confusion matrix: provides detailed insight into classification outcomes, including true positives, true negatives, false positives, and false negatives.

Together, these metrics enabled a comprehensive evaluation of model performance and generalization capability (22, 23). During model evaluation, missing labels were masked and excluded from F1-score and ROC-AUC calculations to ensure reliable performance measurement.

### 2.6 Ethical approval

This study was approved by the Standing Committee of Bioethics Research (SCBR), Deanship of Scientific Research, Prince Sattam Bin Abdulaziz University (PSAU) (Approval No. SCBR-189/2023). The analysis was conducted using anonymized laboratory data collected as part of routine clinical care, and all patient identifiers were removed prior to data processing.

## 3 Result

### 3.1 Data records

Among 18,003 collected reports, *Klebsiella spp*. accounted for 2,646 isolates (13.7%). Of the total cases analyzed, 1,276 (48.2%) were female and 1,015 (38.4%) were male; gender was not reported in 355 (13.4%). Urine was the most frequent source of *Klebsiella spp*. isolates 1,205 (45.5%), followed by blood cultures 408 (15.4%), respiratory samples 370 (14.0%), and wound specimens 324 (12.2%) (Table S4).

Among the 2,646 *Klebsiella spp*. isolates identified, *K. pneumoniae* was the predominant species, accounting for 2,536 (95.8%). This was followed by *K. oxytoca* with 83 (3.1%). Other species were detected at much lower frequencies, including *K. ozaenae* 18 (0.7%), *Klebsiella sp*. 7 (0.3%), and *K. rhinoscleromatis* 2 (0.1%).

### 3.2 Antimicrobial susceptibility testing

**Figure 2:**
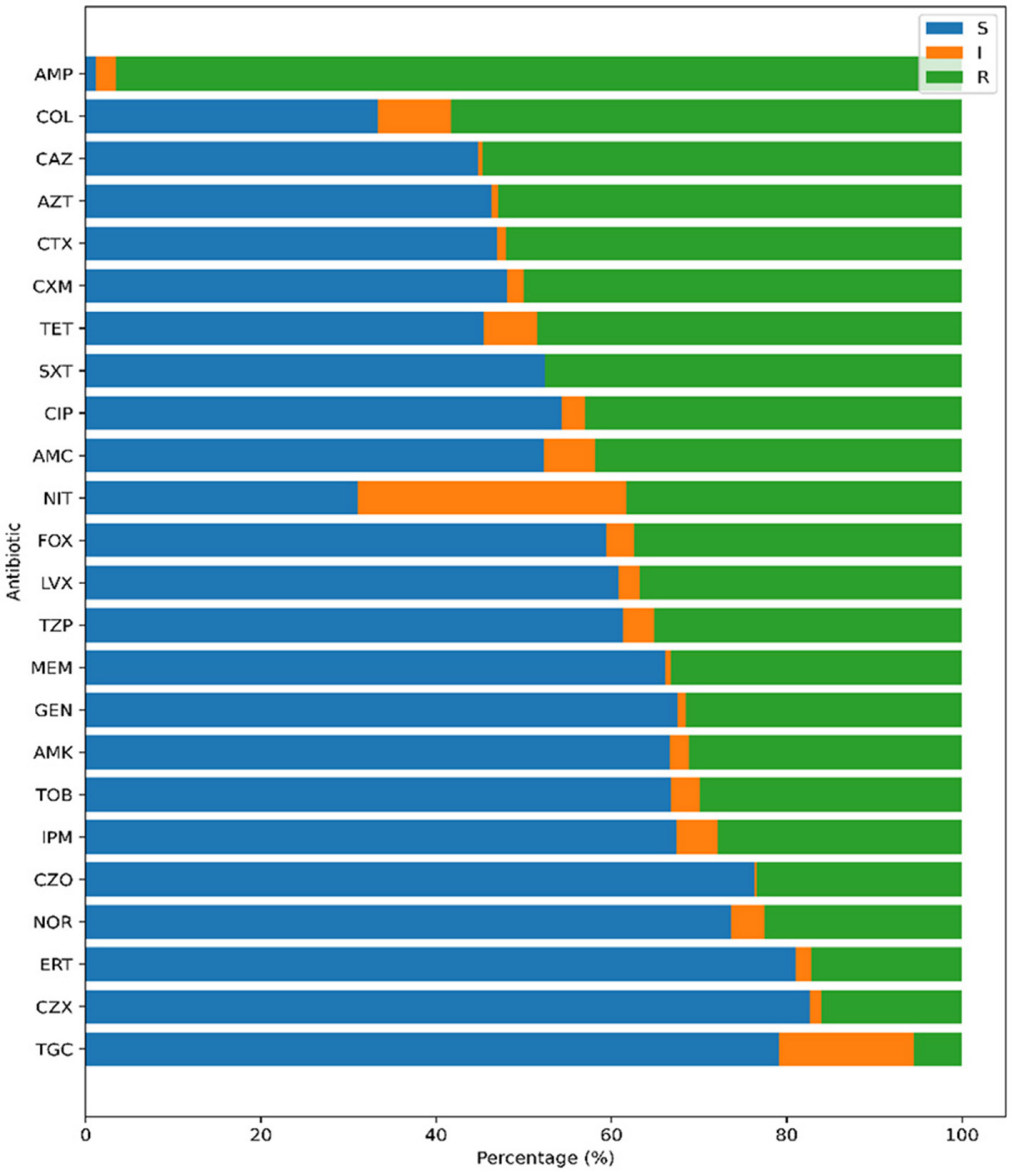
Horizontal stacked bars illustrate the percentage of *Klebsiella pneumoniae* isolates classified as susceptible **(S)**, intermediate **(I)**, and resistant **(R)**, for each tested antibiotic.

Resistance rates varied across Klebsiella species, antibiotics were categorized based on resistance rates into high (≥50%), medium (20%−49%), and low (< 20%) resistance groups. As expected, ampicillin exhibited a high level of non-susceptibility among *Klebsiella* isolates, consistent with the intrinsic resistance of *Klebsiella* spp. mediated by chromosomally encoded β-lactamases. High resistance rates were observed in ceftazidime (54.7%), aztreonam (52.9%), cefotaxime (52.1%), and trimethoprim-sulfamethoxazole (47.6%). Resistance to carbapenems was substantial, with resistance rates of 33.2%, 27.8%, and 17.2% for meropenem, imipenem, and ertapenem, respectively, indicating a substantial prevalence of carbapenem-resistant *Klebsiella* spp. (Figure 2). Colistin susceptibility data were available for only 12 isolates. Among these, 7 isolates were resistant, corresponding to a resistance rate of 58.3% (7/12). Because 99.5% (2,634/2,646) of isolates lacked colistin susceptibility data, this percentage should be interpreted cautiously and may not represent the true prevalence of colistin resistance among all *Klebsiella* isolates (Table S5). While *K. oxytoca* demonstrated lower resistance rates overall (Table S6). The resistance rates of the 15 antibiotics selected for machine learning prediction are shown in Figure S2.

### 3.3 Definitions of resistance phenotypes

Among *Klebsiella* spp. isolates, 57.8% were MDR, ≈ 1.9% were XDR, and No PDR *Klebsiella* spp. isolates were identified in this study. Based on resistance to cefotaxime, ceftazidime, and aztreonam, the estimated prevalence of ESBL-producing isolates was approximately 52%−55%.

### 3.4 Model performance

**Figure 3:**
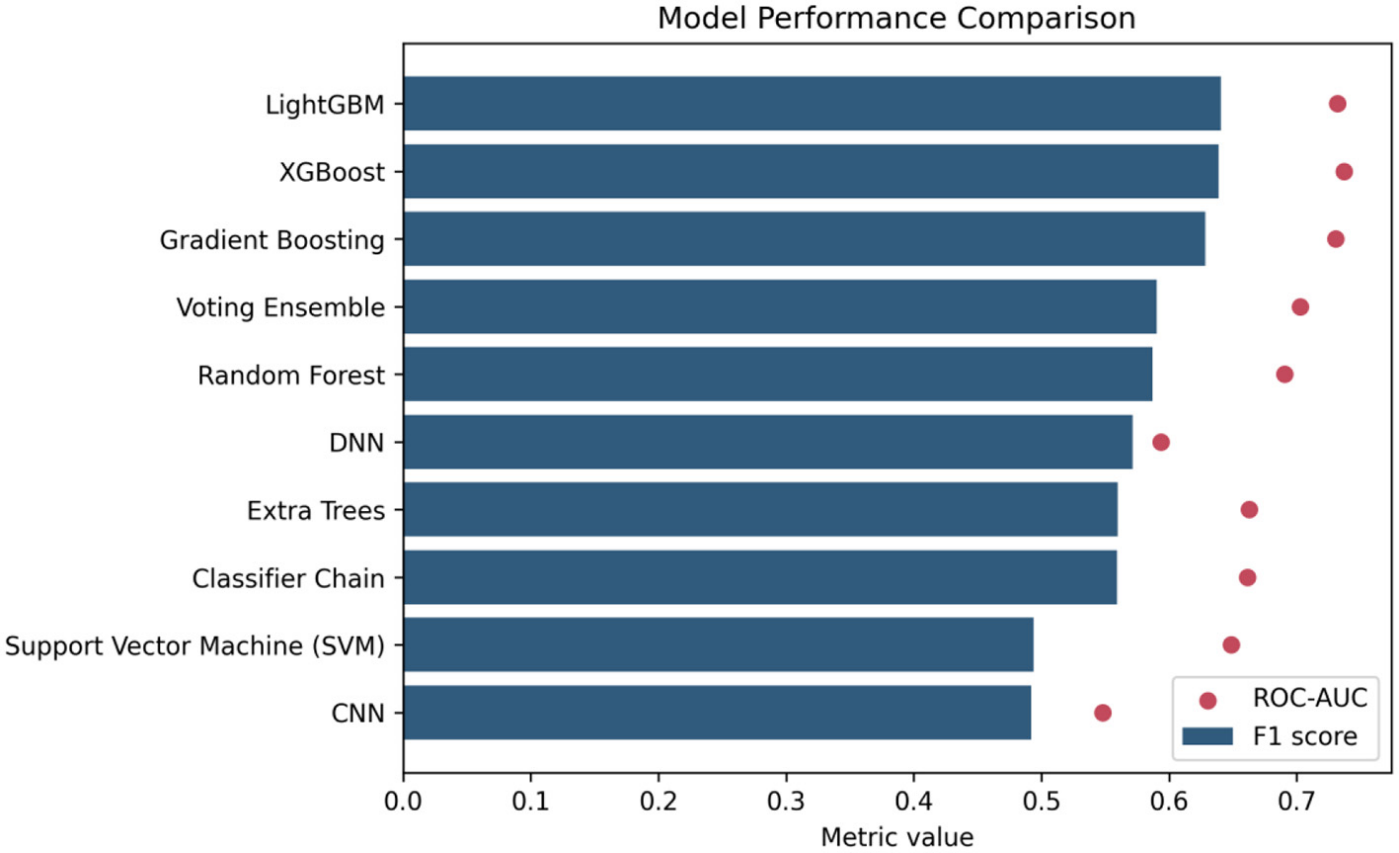
Comparison of machine learning model performance across different algorithms using F1-score and ROC-AUC metrics. The horizontal bars represent the F1-scores of each model, while the red dots indicate the corresponding ROC-AUC values. Ensemble-based methods, including LightGBM, XGBoost, Gradient Boosting, and Voting Ensemble, demonstrate superior overall performance compared to deep learning and traditional machine learning models, with consistently higher F1-scores and ROC-AUC values.

Model performance was evaluated using accuracy, precision, recall, F1-score, and ROC-AUC metrics (Table 1, Figure 3). Overall, gradient boosting-based algorithms demonstrated superior predictive performance compared with other machine learning and deep learning approaches.

**Table 1:** Machine learning model performance comparison.

Model	Accuracy	Precision	Recall	F1 score	ROC-AUC
LightGBM	0.69	0.64	0.65	0.64	0.73
XGBoost	0.70	0.650	0.64	0.64	0.74
Gradient boosting	0.69	0.640	0.63	0.63	0.73
Voting ensemble	0.68	0.623	0.56	0.59	0.70
Random forest	0.66	0.602	0.57	0.59	0.69
DNN	0.55	0.48	0.72	0.57	0.59
Extra trees	0.63	0.57	0.55	0.56	0.66
Classifier chain	0.65	0.57	0.58	0.56	0.66
Support Vector Machine (SVM)	0.64	0.54	0.49	0.49	0.65
CNN	0.51	0.45	0.63	0.49	0.53

Performance varied notably between antibiotics. High-performing predictions were observed for IPM and MEM, with ROC-AUC values of 0.85, indicating strong discriminative ability (Table 2). In contrast, antibiotics such as TGC, AZT, and CTX showed comparatively weaker performance, particularly in terms of precision and overall F1-score, suggesting greater difficulty in accurately modeling resistance patterns for these agents. Notably, SVM and deep neural networks were selected as best models for specific antibiotics (e.g., CXM and AZT), but generally showed lower or inconsistent performance compared to boosting approaches. Overall, the results highlight that no single model is universally optimal, and antibiotic-specific modeling yields the most reliable predictive performance.

**Table 2:** Best machine learning model per antibiotic.

Antibiotic	Best model	Sample count	Accuracy	Precision	Recall	F1-score	ROC-AUC
AMK	XGBoost	5,130	0.73	0.57	0.73	0.64	0.77
GEN	XGBoost	5,120	0.71	0.55	0.63	0.59	0.73
MEM	Gradient boosting	5,070	0.78	0.64	0.80	0.71	0.84
AMC	XGBoost	5,080	0.70	0.71	0.65	0.68	0.75
IPM	XGBoost	5,100	0.79	0.65	0.82	0.72	0.85
CIP	Gradient boosting	5,050	0.74	0.73	0.69	0.71	0.78
SXT	Classifier chain	5,030	0.61	0.56	0.77	0.65	0.64
TZP	XGBoost	4,960	0.77	0.70	0.73	0.72	0.81
CXM	Support Vector Machine (SVM)	4,930	0.62	0.64	0.82	0.72	0.61
CAZ	LightGBM	4,850	0.69	0.77	0.65	0.70	0.72
LVX	XGBoost	4,890	0.76	0.68	0.71	0.69	0.82
FOX	LightGBM	4,420	0.70	0.65	0.60	0.63	0.70
AZT	DNN	4,410	0.58	0.59	0.792	0.67	0.56
CTX	Classifier chain	4,010	0.58	0.60	0.679	0.64	0.62
TGC	LightGBM	3,860	0.64	0.28	0.458	0.35	0.57

### 3.5 ROC curve analysis

**Figure 4:**
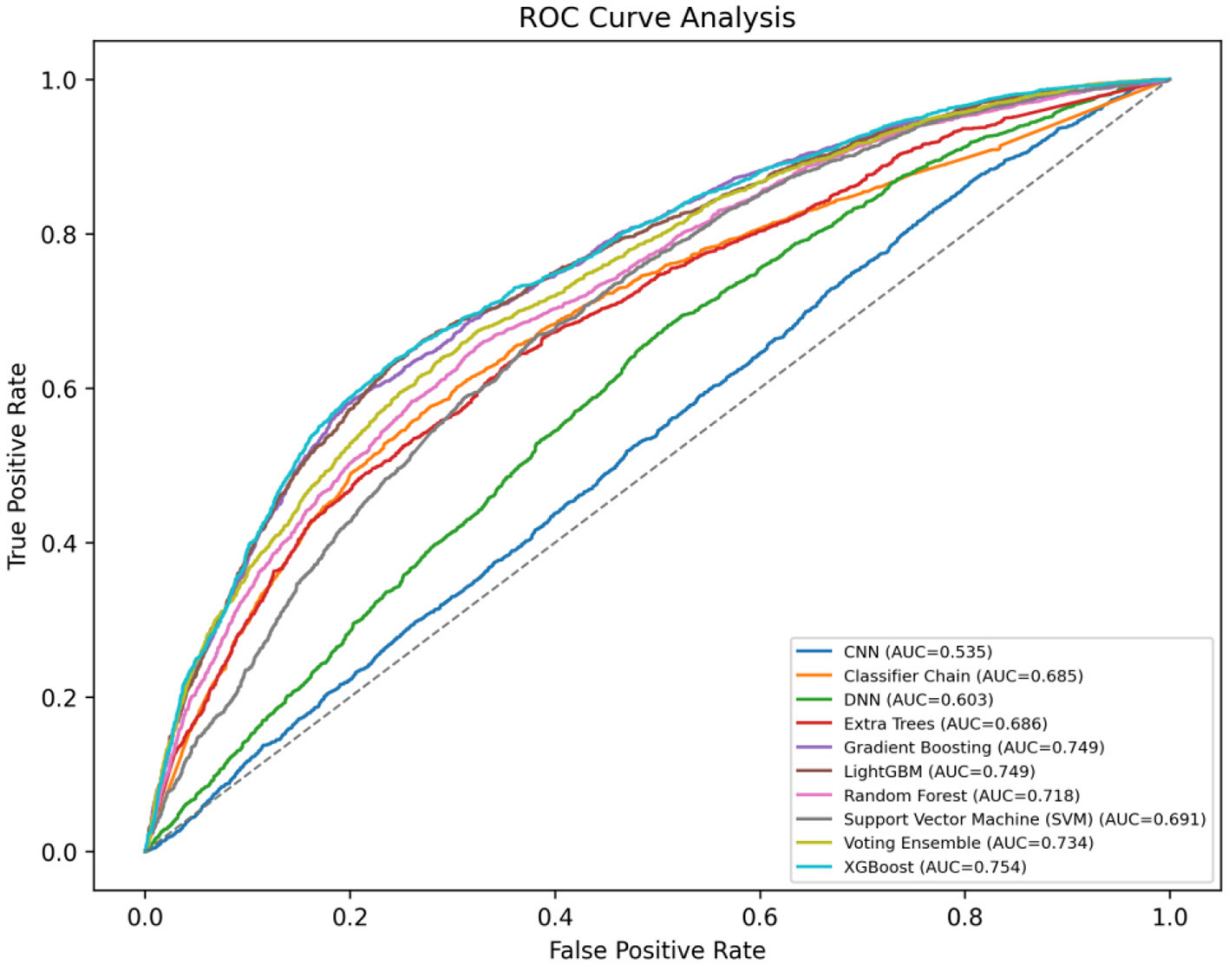
Receiver operating characteristic (ROC) curves comparing the predictive performance of multiple machine learning models, including Random Forest, Support Vector Machine (SVM), Gradient Boosting, XGBoost, LightGBM, Extra Trees, Voting Ensemble, Deep Neural Network (DNN), and Convolutional Neural Network (CNN).

The ROC curves demonstrate strong classification performance across all models, with the XGBoost exhibiting the highest discriminative ability (Figure 4).

### 3.6 Feature importance

Feature importance analysis revealed that clinical context (source) and time-related variables (month/year) are the primary drivers of model behavior, while demographic and microbiological identifiers play secondary roles. A potential methodological consideration is feature redundancy among temporal variables (month, quarter, year), which may distribute importance unevenly and slightly obscure the true strength of seasonal effects. These results are provided in the (Table S3, Figure S3).

### 3.7 Confusion matrix

Antibiotic-specific confusion matrices were generated for all tested antibiotics, the combined summary is shown in (Table 3). Model performance varied substantially depending on the antibiotic type and dataset characteristics. Accuracy values ranged from 0.58 (CTX and AZT) to 0.79 (IPM), indicating moderate to good predictive capability across most antibiotics. Similarly, recall values showed generally strong sensitivity in detecting positive cases for several antibiotics, particularly IPM (0.82), CXM (0.82), and AMK (0.73), suggesting that the models were effective in identifying resistant or non-susceptible isolates in these cases. However, specificity showed wider variability, with notably lower values for CXM (0.30) and AZT (0.33), indicating a higher rate of false positives for these antibiotics.

**Table 3:** Confusion matrix summary for all tested antibiotics.

Antibiotic	Best model	TN	FP	FN	TP	Total	Accuracy	Recall	Specificity
AMK	XGBoost	249	94	46	124	513	0.73	0.73	0.73
GEN	XGBoost	260	86	61	105	512	0.71	0.63	0.75
MEM	Gradient boosting	259	77	34	137	507	0.78	0.80	0.77
AMC	XGBoost	197	65	86	160	508	0.70	0.65	0.75
IPM	XGBoost	258	77	32	143	510	0.79	0.82	0.77
CIP	Gradient boosting	216	60	71	158	505	0.74	0.69	0.78
SXT	Classifier chain	120	144	54	185	503	0.61	0.77	0.46
TZP	XGBoost	235	62	53	146	496	0.77	0.73	0.79
CXM	SVM	58	136	54	245	493	0.62	0.82	0.30
CAZ	LightGBM	159	54	95	177	485	0.69	0.65	0.75
LVX	XGBoost	235	63	56	135	489	0.76	0.71	0.79
FOX	LightGBM	198	60	73	111	442	0.70	0.60	0.77
AZT	DNN	67	134	50	190	441	0.58	0.79	0.33
CTX	Classifier chain	85	98	70	148	401	0.58	0.68	0.47
TGC	LightGBM	207	96	45	38	386	0.64	0.46	0.68

TN, true negative: number of correctly predicted susceptible cases (the model correctly predicts the isolate is not resistant); FP, false positive: number of cases incorrectly predicted as resistant when they are actually susceptible. FN, false negative: number of cases incorrectly predicted as susceptible when they are actually resistant (clinically the most critical error in many antibiotic studies); TP, true positive: number of correctly predicted resistant cases (the model correctly identifies resistance).

### 3.8 Prediction output

The trained model was further evaluated using unseen test data to assess its real-world predictive performance. Predictions were generated for each antibiotic, including probability-based classification outputs for resistant and susceptible profiles. A comparison between the actual and predicted antibiotic susceptibility results for representative test samples is presented in Table 4. For most antibiotics, the selected models (including XGBoost, Gradient Boosting, LightGBM, SVM, DNN, and Classifier Chain) were able to correctly identify resistant isolates with relatively high predicted probabilities, indicating strong discriminative capability. However, several misclassifications are also observed, particularly in borderline cases where susceptible isolates were incorrectly predicted as resistant or vice versa. These errors are often associated with intermediate probability values close to the decision threshold, highlighting the inherent uncertainty in classification for some samples.

**Table 4:** Prediction comparison examples using the best model for each antibiotic.

Sample index	Antibiotic	Best model	Actual label	Predicted label text	Predicted probability resistant
0	AMK	XGBoost	S	R	0.654
2	AMK	XGBoost	S	R	0.774
19	AMK	XGBoost	R	R	0.668
0	GEN	XGBoost	S	S	0.234
2	GEN	XGBoost	S	S	0.293
19	GEN	XGBoost	S	S	0.115
2	MEM	Gradient boosting	R	R	0.819
19	MEM	Gradient boosting	R	R	0.844
28	MEM	Gradient boosting	S	R	0.755
0	AMC	XGBoost	S	R	0.749
19	AMC	XGBoost	R	R	0.755
24	AMC	XGBoost	S	R	0.776
2	IPM	XGBoost	R	R	0.866
19	IPM	XGBoost	R	R	0.850
28	IPM	XGBoost	S	R	0.844
0	CIP	Gradient boosting	S	R	0.839
19	CIP	Gradient boosting	R	R	0.827
24	CIP	Gradient boosting	S	R	0.820
0	SXT	Classifier chain	S	R	0.500
2	SXT	Classifier chain	S	R	0.628
19	SXT	Classifier chain	R	R	0.780
2	TZP	XGBoost	R	R	0.748
43	TZP	XGBoost	R	R	0.732
50	TZP	XGBoost	S	R	0.687
0	CXM	SVM	S	R	0.707
2	CXM	SVM	R	R	0.697
19	CXM	SVM	R	R	0.672
0	CAZ	LightGBM	S	R	0.863
2	CAZ	LightGBM	R	R	0.898
28	CAZ	LightGBM	R	R	0.829
62	LVX	XGBoost	R	R	0.672
68	LVX	XGBoost	S	R	0.571
79	LVX	XGBoost	S	R	0.571
0	FOX	LightGBM	S	R	0.865
2	FOX	LightGBM	R	R	0.951
50	FOX	LightGBM	R	R	0.699
36	AZT	DNN	S	R	0.586
50	AZT	DNN	S	R	0.540
62	AZT	DNN	R	R	0.532
0	CTX	Classifier chain	S	R	0.952
19	CTX	Classifier chain	R	R	1.000
50	CTX	Classifier chain	S	R	0.519
19	TGC	LightGBM	S	S	0.139
33	TGC	LightGBM	S	S	0.197
38	TGC	LightGBM	S	S	0.197

Notably, antibiotics such as IPM, MEM, and CAZ show more confident predictions for resistant cases (higher predicted probabilities), whereas antibiotics like SXT and TGC demonstrate greater variability in probability outputs, reflecting more challenging classification patterns.

Overall, this table provides a case-level validation of the models, demonstrating not only their predictive strength but also the nature of their misclassifications, which is important for understanding model reliability in clinical decision-support contexts.

## 4 Discussion

In this study, *Klebsiella* species constituted a notable proportion of clinical isolates, accounting for 2,646 (13.7%) of the 18,003 microbiology reports analyzed over the five-year period. This considerable representation reflects the substantial clinical burden of *Klebsiella* infections in healthcare settings. The finding is consistent with previous reports that identify *Klebsiella* spp. as important opportunistic pathogens responsible for both community-acquired and hospital-associated infections (24–26). The relatively high prevalence observed further emphasizes the growing relevance of *Klebsiella* species in antimicrobial resistance surveillance and infection control efforts. Regarding patient demographic, a slightly higher proportion of isolates was obtained from female patients (48.2%) compared with male patients (38.4%), while gender information was unavailable for 13.4% of cases. This distribution may reflect the predominance of urinary tract infections within the dataset, which are generally more common among females due to anatomical and physiological factors. Such a pattern is consistent with previous epidemiological studies reporting increased susceptibility of female patients to urinary tract infections caused by *Klebsiella* species (27).

The distribution of specimen sources further supports this interpretation. Urine samples accounted for the largest proportion of *Klebsiella* isolates (45.5%), followed by blood cultures (15.4%), respiratory samples (14.0%), and wound specimens (12.2%). The predominance of urinary isolates reflects the well-established role of *Klebsiella* species as leading pathogens in urinary tract infections, particularly among hospitalized patients and those with catheter-associated infections. The substantial proportion of bloodstream isolates underscores the potential severity of *Klebsiella* infections, as bacteremia and sepsis caused by *Klebsiella* spp. are frequently associated with increased morbidity and mortality. In addition, the presence of isolates from wound and respiratory specimens highlights the opportunistic nature of these pathogens, particularly in immunocompromised individuals and patients with prolonged hospital stays (28, 29).

Overall, the predominance of *K. pneumoniae*, together with its high isolation rate from urine and bloodstream samples, underscores its clinical significance. These findings reinforce the need for continuous surveillance, targeted antimicrobial stewardship programs, and strengthened infection control strategies to limit the spread of resistant *Klebsiella* strains in healthcare settings.

The present study also revealed heterogeneous resistance patterns among *Klebsiella* isolates. Ampicillin (96.6%) and ceftazidime (54.7 %) exhibited the highest resistance rates, indicating limited clinical utility. The high level of ampicillin resistance observed in this study is consistent with the well-established intrinsic resistance of *Klebsiella* spp., which naturally produce chromosomally encoded β-lactamases capable of hydrolyzing ampicillin. Therefore, this finding should be interpreted as an inherent biological characteristic of the genus rather than an acquired resistance trend, whereas the high resistance to ceftazidime likely reflects the growing prevalence of extended-spectrum β-lactamase (ESBL)-producing strains, as reported globally and within Saudi Arabia (28, 29). Several antibiotics demonstrated moderate resistance levels, including trimethoprim–sulfamethoxazole, aztreonam, ciprofloxacin, amoxicillin–clavulanate, cefuroxime, cefotaxime, levofloxacin, and piperacillin–tazobactam. These findings are consistent with regional reports documenting increasing resistance to fluoroquinolones and β-lactams among *K. pneumoniae* isolates (27, 29). A high proportion of isolates exhibited non-susceptibility to carbapenems (meropenem 33.2% and imipenem 27.8%), indicating a concerning level of carbapenem resistance within the study population. (28, 30). In contrast, low resistance rates were observed for tigecycline, tetracycline, ertapenem, and norfloxacin, indicating the retained effectiveness of these agents. Collectively, these findings align with previous studies and underscore the growing resistance among *Klebsiella* species, highlighting the importance of continuous surveillance and strengthened antimicrobial stewardship efforts.

The multidrug-resistant (MDR) rate observed in this study (57.8%) aligns with previous reports from Saudi Arabia and other regions, where MDR *K. pneumoniae* prevalence has ranged from 40 to 70% (28, 31–33). Similarly, studies conducted across Gulf countries have documented the widespread dissemination of MDR *K. pneumoniae*, along with the emergence of carbapenemase-producing strains. These developments are particularly concerning, as they are associated with limited therapeutic options and increased morbidity and mortality (34). The extensively drug-resistant (XDR) rate of 1.9% identified in this study is inconsistent with previously published data, which report XDR prevalence ranging from 5 to 20% among *K. pneumoniae* isolate (35). The presence of XDR strains is especially alarming, given their resistance to most available antimicrobial agents and the resulting challenges in clinical management, often accompanied by poorer patient outcomes.

Despite the elevated MDR and XDR rates, no pan-drug-resistant (PDR) isolates were identified. This finding is in line with earlier studies indicating that pan-drug resistance in *K. pneumoniae* remains relatively uncommon. Nevertheless, the observed resistance patterns underscore the growing burden of antimicrobial resistance and reinforce the need for ongoing surveillance and strengthened antimicrobial stewardship efforts.

The present study demonstrated that among all evaluated models, XGBoost achieved the best overall performance, with an accuracy of 0.70, precision of 0.65, recall of 0.64, an F1-score of 0.64, and a ROC-AUC of 0.74. LightGBM showed comparable performance, yielding an F1-score of 0.64 and a ROC-AUC of 0.73, followed closely by Gradient Boosting, which achieved an F1-score of 0.63 and a ROC-AUC of 0.73. Although the Voting Ensemble model exhibited a relatively high ROC-AUC of 0.70, its F1-score was lower (0.59) compared with the top-performing models. Similarly, Random Forest demonstrated moderate performance, with an F1-score of 0.59 and a ROC-AUC of 0.69. Among the remaining machine learning approaches, Extra Trees and Classifier Chain achieved comparable performance, with F1-scores of 0.56 and 0.56 and ROC-AUC values of 0.66 and 0.66, respectively. The Support Vector Machine (SVM) exhibited lower predictive capability, producing an F1-score of 0.49 and a ROC-AUC of 0.65. Deep learning models showed mixed performance. The Deep Neural Network (DNN) achieved an F1-score of 0.57 and a ROC-AUC of 0.59, outperforming the Convolutional Neural Network (CNN), which yielded the lowest overall performance, with an F1-score of 0.49 and a ROC-AUC of 0.55. Notably, the DNN achieved the highest recall value (0.72), indicating improved sensitivity despite lower overall discriminative performance. Recent investigations have similarly shown that XGBoost approaches achieve strong predictive performance for antibiotic resistance when applied to large-scale surveillance datasets (36, 37). The application of machine learning to antimicrobial resistance prediction has shown promising results in different healthcare settings. A study from Jordan utilized CART and Random Forest models to predict antimicrobial resistance using microbiological and demographic data from 2,893 clinical reports. Random Forest achieved superior predictive performance, with accuracies ranging from 0.64 to 0.99, and identified demographic factors and bacterial species as significant contributors to resistance prediction (38). These findings are consistent with the growing evidence that machine learning algorithms can effectively identify resistance patterns and support antimicrobial stewardship efforts when used alongside conventional microbiological testing.

In addition, systematic reviews of machine learning applications in antimicrobial resistance prediction indicate that ensemble learning methods consistently deliver improved accuracy and generalization, supporting their suitability for clinical decision support systems (39, 40). In the present study, our findings indicate that boosting-based machine learning algorithms, particularly XGBoost, LightGBM, and Gradient Boosting, provided the most reliable predictive performance for antibiotic resistance prediction, outperforming ensemble tree-based methods and deep learning approaches. The high F1-score observed reflects a balanced performance between precision and recall, which is particularly important in clinical applications where both false positives and false negatives carry significant consequences. Such performance suggests that the model may serve as an adjunctive decision-support tool by providing early estimates of antimicrobial susceptibility patterns while conventional laboratory results are pending. However, these predictions are not intended to replace phenotypic or genotypic antimicrobial susceptibility testing, which remains essential for definitive clinical decision-making.

The comparison between actual and model-predicted antibiotic resistance profiles for selected test samples using the best model for each antibiotic further demonstrated its predictive capability. These models effectively captured complex resistance patterns and maintained strong predictive performance across multiple antibiotics simultaneously. The high level of agreement between observed and predicted results reinforces the reliability and potential clinical applicability of the proposed machine learning approach. These results highlight that no single model uniformly performs best across all antibiotics, emphasizing the importance of antibiotic-specific model optimization. The observed variability also suggests that resistance prediction is highly dependent on both antibiotic mechanism and data distribution, supporting the use of tailored machine learning pipelines rather than a universal predictive model.

Overall, boosting-based machine learning algorithms consistently achieved the highest predictive performance, with XGBoost, LightGBM, and Gradient Boosting identified as the best-performing models. Such models may support integration into clinical decision-support systems and antimicrobial stewardship programs, ultimately improving patient outcomes and optimizing antibiotic use.

## 5 Conclusion

This study underscores the substantial clinical burden of *Klebsiella* infections, with *Klebsiella* spp. accounting for 13.7% of clinical isolates over the five-year period, predominantly *K. pneumoniae*. Urinary tract infections represented the most common source, followed by bloodstream, wound, and respiratory infections, highlighting the opportunistic behavior and clinical significance of *Klebsiella* species. In addition, high resistance rates to commonly used antibiotics particularly ceftazidime along with moderate resistance to several β-lactams and fluoroquinolones, reflect the growing challenge of antimicrobial resistance. Although the last-line agents such as colistin and tigecycline remained largely effective, emerging resistance trends remain concerning. The findings demonstrate a substantial burden of carbapenem-resistant *Klebsiella* spp., underscoring a critical antimicrobial resistance challenge and the urgent need for strengthened antimicrobial stewardship and continuous surveillance, and the implementation of advanced predictive tools to limit the spread of resistant *Klebsiella* strains and improve patient outcomes.

Also, this study presents an effective machine learning-based framework for predicting antimicrobial resistance. The boosting-based machine learning algorithms achieved the best performance and demonstrated strong predictive capability. The approach can be extended to real-world clinical applications for improved patient outcomes.

### 5.1 Study limitation

Despite its strengths, this study has several limitations that should be considered when interpreting the findings; the dataset contained missing values for several antibiotics, necessitating the selection of agents with lower levels of missing data. Although this approach improved model reliability, it may have reduced the overall comprehensiveness of the analysis. Second, the study was conducted within a single geographic area, which may limit the generalizability of the model to other populations, healthcare settings, or regions.

In addition, the model did not incorporate temporal or longitudinal data, which could potentially enhance predictive accuracy by capturing evolving resistance patterns over time. Also, grouping of intermediate and resistant isolates into a single non-susceptible category. Although this approach improved class balance and model stability, it may obscure biological and clinical differences between intermediate and fully resistant phenotypes. Future studies with larger datasets should evaluate multiclass classification models that distinguish susceptible, intermediate, and resistant isolates. Finally, the current framework relied solely on structured clinical data and did not include genomic or molecular features. The integration of such data in future studies may further improve predictive performance and provide deeper insights into the mechanisms underlying antimicrobial resistance.

## Data Availability

The original contributions presented in the study are included in the article/Supplementary material, further inquiries can be directed to the corresponding author.
