# Supplementary material for: Machine learning based prediction of antimicrobial resistance in Klebsiella spp.: a five-year retrospective study
Source: Front Public Health. 2026 Jul 1;14:1865551. doi: 10.3389/fpubh.2026.1865551 (PMC13403328; doi:10.3389/fpubh.2026.1865551)
Supplement: Supplementary file 1 [file Supplementary_file_1.docx]

**Supplementary Data**

**Machine Learning Based Prediction of Antimicrobial Resistance *in Klebsiella spp*.: A Five-Year Retrospective Study**

**Table S1. Antimicrobial agents included in the study and their classification by antimicrobial class**

| Antimicrobial class | Antibiotic | Abbreviation |
| --- | --- | --- |
| Aminoglycosides | Amikacin | AMK |
|  | Gentamicin | GEN |
|  | Tobramycin | TOB |
| Penicillins | Ampicillin | AMP |
| β-Lactam/β-Lactamase Inhibitor Combinations | Amoxicillin–Clavulanate | AMC |
|  | Piperacillin–Tazobactam | TZP |
| Cephalosporins | Cefazolin | CZO |
|  | Cefotaxime | CTX |
|  | Ceftriaxone | CZX |
|  | Cefoxitin | FOX |
|  | Ceftazidime | CAZ |
|  | Cefuroxime | CXM |
| Carbapenems | Ertapenem | ERT |
|  | Imipenem | IPM |
|  | Meropenem | MEM |
| Monobactams | Aztreonam | AZT |
| Fluoroquinolones | Ciprofloxacin | CIP |
|  | Levofloxacin | LVX |
|  | Norfloxacin | NOR |
| Folate Pathway Inhibitors | Trimethoprim–Sulfamethoxazole | SXT |
| Tetracyclines / Glycylcyclines | Tetracycline | TET |
|  | Tigecycline | TGC |
| Polymyxins | Colistin | COL |
| Nitrofuran Derivatives | Nitrofurantoin | NIT |

**Table S2: Antibiotics used for ML prediction and Missing percentage**

| **Antibiotics** | **Missing** | **Missing %** | **Status** | **Justification** |
| --- | --- | --- | --- | --- |
| AMK | 91 | 3.44 | Included | Missing percentage <=26% |
| AMC | 124 | 4.69 | Included | Missing percentage <=26% |
| AMP | 152 | 5.74 | Excluded | Intrinsic Resistance |
| AZT | 397 | 15 | Included | Missing percentage <=26% |
| CZO | 1297 | 49.02 | Excluded | >26% Missing |
| CZX | 1208 | 45.65 | Excluded | >26% Missing |
| CTX | 584 | 22.07 | Included | Missing percentage <=26% |
| FOX | 382 | 14.44 | Included | Missing percentage <=26% |
| CAZ | 211 | 7.97 | Included | Missing percentage <=26% |
| CXM | 181 | 6.84 | Included | Missing percentage <=26% |
| CIP | 138 | 5.22 | Included | Missing percentage <=26% |
| COL | 2634 | 99.55 | Excluded | >26% Missing |
| ERT | 1208 | 45.65 | Excluded | >26% Missing |
| GEN | 97 | 3.67 | Included | Missing percentage <=26% |
| IPM | 127 | 4.8 | Included | Missing percentage <=26% |
| LVX | 237 | 8.96 | Included | Missing percentage <=26% |
| MEM | 122 | 4.61 | Included | Missing percentage <=26% |
| NIT | 1465 | 55.37 | Excluded | >26% Missing |
| NOR | 1796 | 67.88 | Excluded | >26% Missing |
| TZP | 180 | 6.8 | Included | Missing percentage <=26% |
| TET | 2613 | 98.75 | Excluded | >26% Missing |
| TGC | 656 | 24.79 | Included | Missing percentage <=26% |
| TOB | 920 | 34.77 | Excluded | >26% Missing |
| SXT | 139 | 5.25 | Included | Missing percentage <=26% |


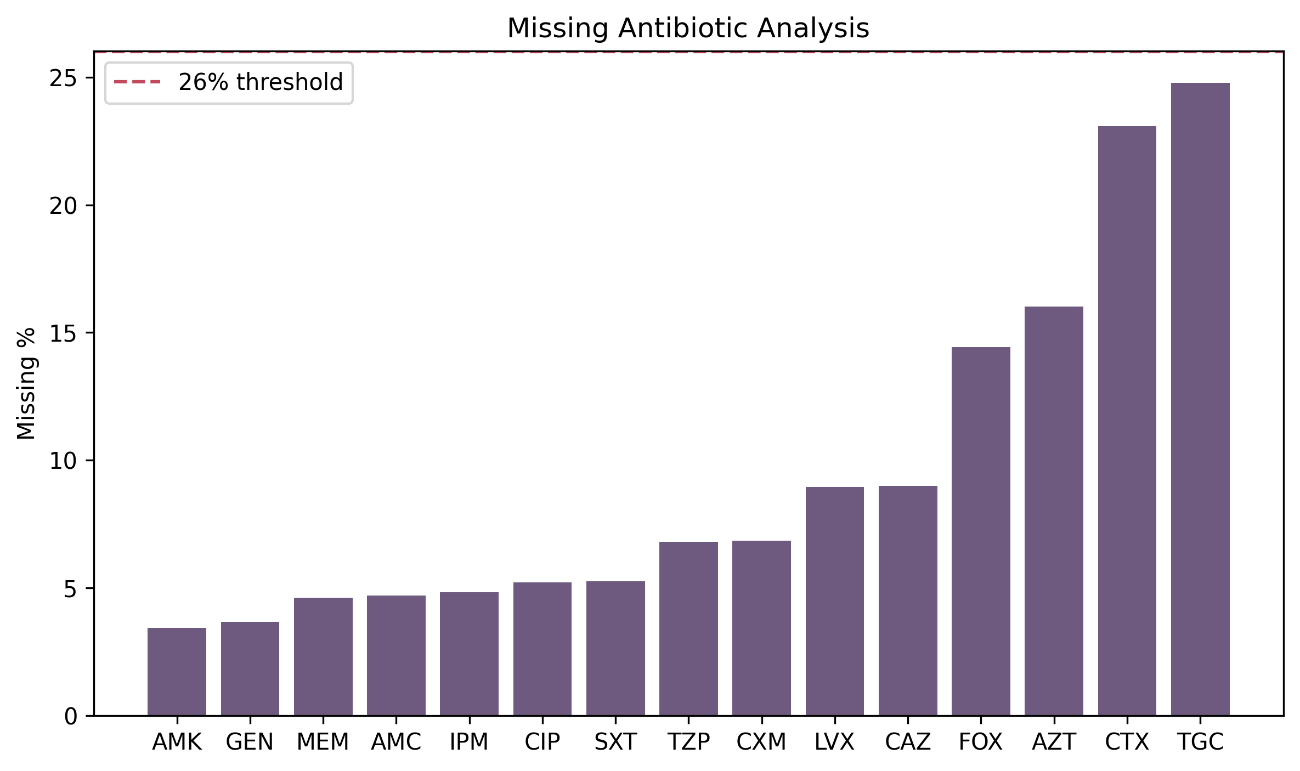


**Figure S1: Missing data percentage per antibiotics**

**Table S3. Ranked feature importance summary across tree-based models.**

| **Rank** | **Feature** | **Mean Importance** | **Relative Importance%** |
| --- | --- | --- | --- |
| 1 | Source | 0.48 | 48.96 |
| 2 | Month | 0.16 | 16.2 |
| 3 | Year | 0.14 | 14.75 |
| 4 | Gender | 0.10 | 10.62 |
| 5 | Organism Name | 0.05 | 5.23 |
| 6 | Quarter | 0.04 | 4.24 |

**Table S4: Distribution of Klebsiella spp. isolates by grouped specimen source**

| Specimen Source | n (%) |
| --- | --- |
| Urine | 1205 (45.5) |
| Blood | 408 (15.4) |
| Respiratory specimens | 370 (14.0) |
| Wound specimens | 324 (12.2) |
| Abscess/Pus | 86 (3.3) |
| Vaginal swab | 62 (2.3) |
| Not reported | 32 (1.2) |
| Tissue | 27 (1.0) |
| Catheter and tip cultures | 24 (0.9) |
| Fluid specimens | 23 (0.9) |
| Ear swab | 20 (0.8) |
| General swab | 18 (0.7) |
| Eye swab | 9 (0.3) |
| Stool | 8 (0.3) |
| CSF | 4 (0.2) |
| Throat swab | 4 (0.2) |
| Fungal culture | 2 (0.1) |
| Anaerobic culture | 1 (<0.1) |


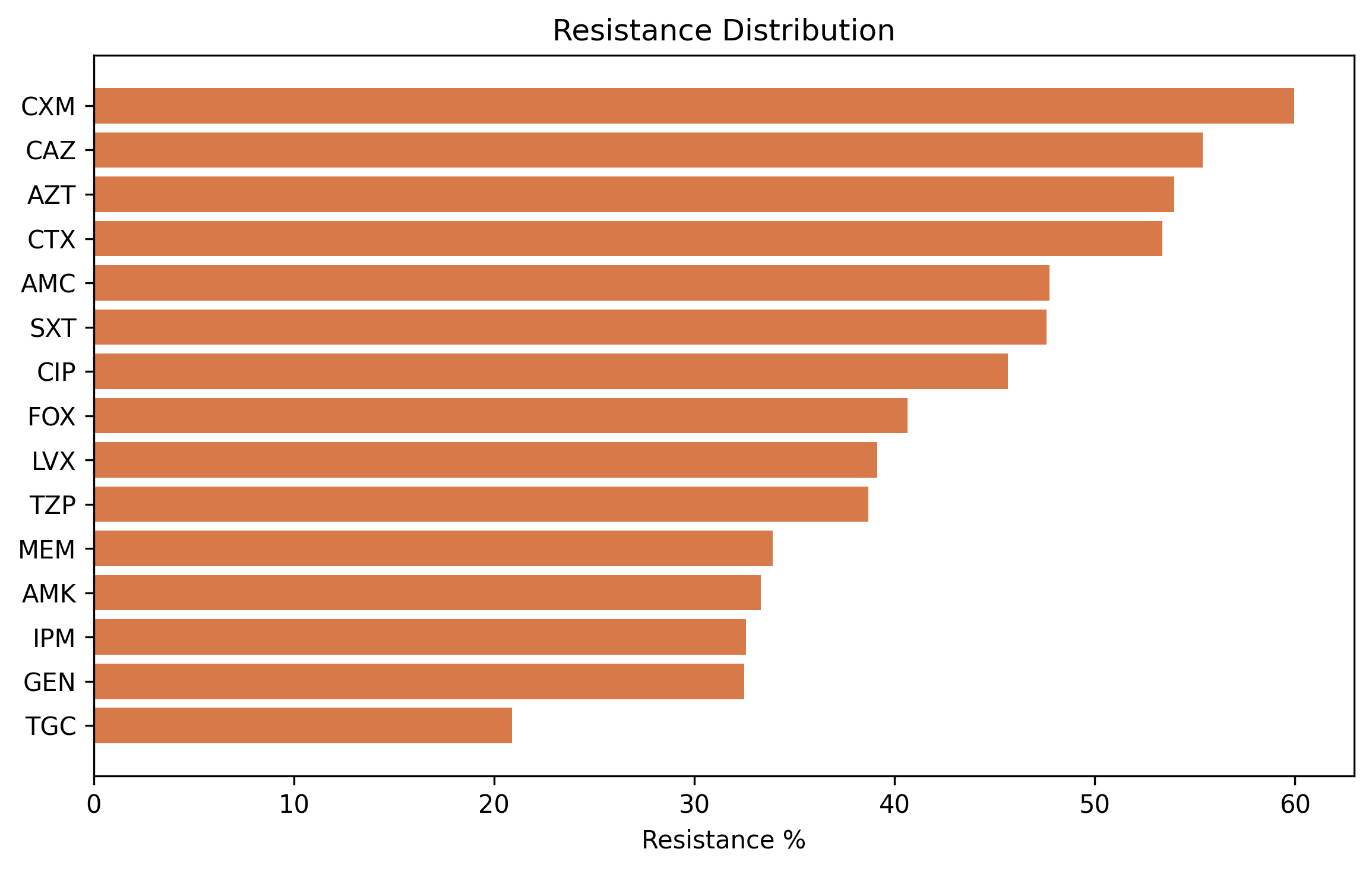


**Figure S2: Distribution of resistance rate among selected antibiotics.**


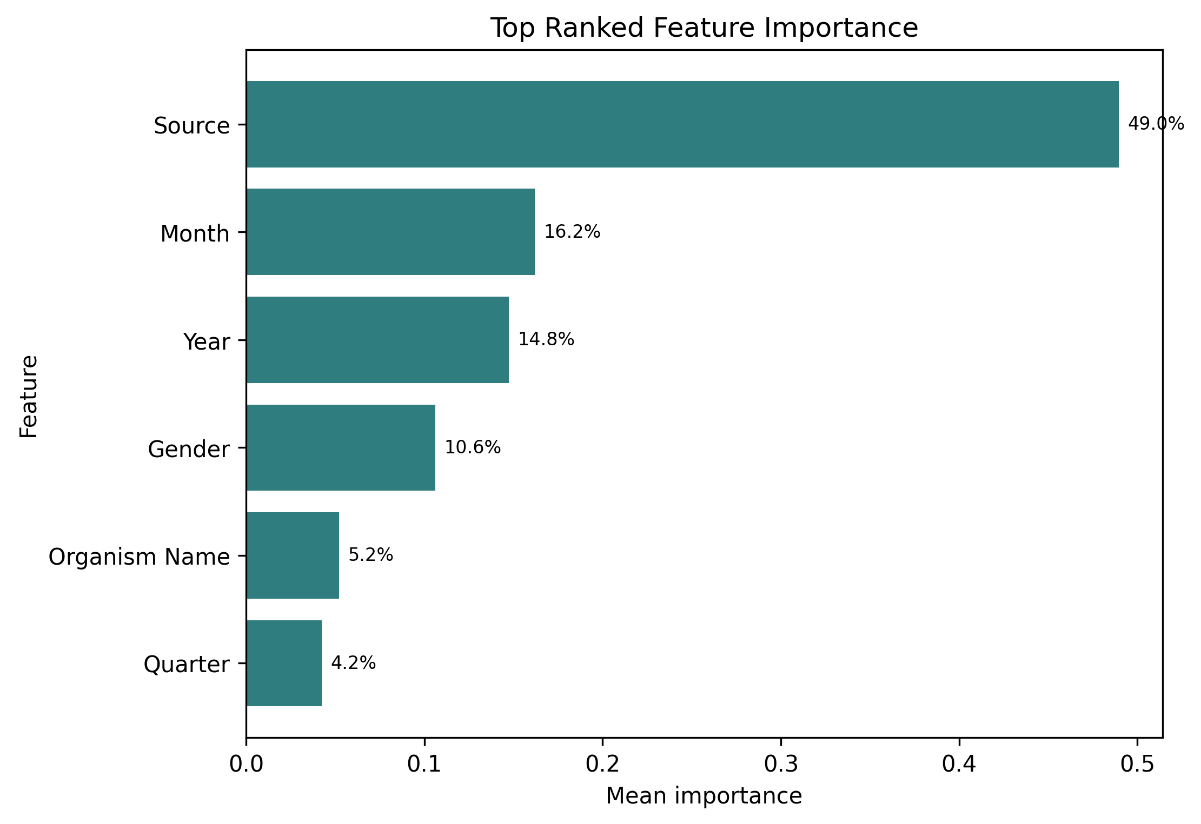


**Figure S3: Feature importance summary.**

**Table S5. Antimicrobial susceptibility results of *Klebsiella* spp.**

| Antibiotic | Tested (n) | S | I | R | Missing | Resistance (%) |
| --- | --- | --- | --- | --- | --- | --- |
| AMK | 2555 | 1704 | 56 | 795 | 91 | 31.12 |
| AMC | 2522 | 1318 | 148 | 1056 | 124 | 41.87 |
| AMP | 2080 | 24 | 47 | 2009 | 566 | 96.59 |
| AZT | 2207 | 1023 | 17 | 1167 | 439 | 52.88 |
| CZO | 806 | 615 | 2 | 189 | 1840 | 23.45 |
| CZX | 1024 | 846 | 14 | 164 | 1622 | 16.02 |
| CTX | 2022 | 949 | 20 | 1053 | 624 | 52.08 |
| FOX | 2264 | 1344 | 73 | 847 | 382 | 37.41 |
| CAZ | 2396 | 1074 | 12 | 1310 | 250 | 54.67 |
| CXM | 2051 | 987 | 38 | 1026 | 595 | 50.02 |
| CIP | 2508 | 1363 | 66 | 1079 | 138 | 43.02 |
| COL | 12 | 4 | 1 | 7 | 2634 | 58.33 |
| ERT | 1437 | 1164 | 26 | 247 | 1209 | 17.19 |
| GEN | 2549 | 1721 | 25 | 803 | 97 | 31.50 |
| IPM | 2518 | 1698 | 119 | 701 | 128 | 27.84 |
| LVX | 2409 | 1466 | 58 | 885 | 237 | 36.74 |
| MEM | 2524 | 1668 | 17 | 839 | 122 | 33.24 |
| NIT | 1181 | 367 | 361 | 453 | 1465 | 38.36 |
| NOR | 850 | 626 | 32 | 192 | 1796 | 22.59 |
| TZP | 2466 | 1512 | 89 | 865 | 180 | 35.08 |
| TET | 33 | 15 | 2 | 16 | 2613 | 48.48 |
| TGC | 1990 | 1574 | 307 | 109 | 656 | 5.48 |
| TOB | 1726 | 1153 | 56 | 517 | 920 | 29.95 |
| SXT | 2507 | 1314 | 0 | 1193 | 139 | 47.59 |

**Table S6: Antibiotics resistant rate for isolated *K. spp.***

| **Organism** | **No.** | **AMK** | **AMC** | **AMP** | **AZT** | **CZO** | **CZX** | **CTX** | **FOX** | **CAZ** | **CXM** | **CIP** | **COL** | **ERT** | **GEN** | **IPM** | **LVX** | **MEM** | **NIT** | **NOR** | **TZP** | **TET** | **TGC** | **TOB** | **SXT** |
| --- | --- | --- | --- | --- | --- | --- | --- | --- | --- | --- | --- | --- | --- | --- | --- | --- | --- | --- | --- | --- | --- | --- | --- | --- | --- |
| *Klebsiella oxytoca* | 83 | 4.82 | 37.35 | 92.65 | 38.16 | 22.73 | 10.42 | 41.67 | 30.26 | 38.27 | 45.45 | 27.71 | NA | 12.90 | 18.07 | 12.05 | 17.07 | 15.66 | 17.39 | 31.43 | 10.84 | NA | 9.86 | 30.16 | 34.94 |
| *Klebsiella ozaenae* | 18 | 72.22 | 76.47 | 93.75 | 66.67 | 0.00 | 0.00 | 66.67 | 70.00 | 71.43 | 73.33 | 76.47 | NA | 0.00 | 0.00 | 75.00 | 69.23 | 75.00 | 100.00 | NA | 75.00 | NA | 0.00 | 0.00 | 70.59 |
| *Klebsiella pneumoniae* | 2,536 | 31.81 | 41.63 | 96.78 | 53.36 | 23.32 | 16.29 | 52.37 | 37.47 | 55.21 | 49.95 | 43.33 | 58.33 | 17.38 | 32.20 | 28.04 | 37.25 | 33.55 | 38.97 | 22.14 | 35.64 | 48.48 | 5.38 | 29.99 | 47.94 |
| *Klebsiella rhinoscleromatis* | 2 | 0.00 | 50.00 | 50.00 | 50.00 | 0.00 | 0.00 | 0.00 | 50.00 | 50.00 | 50.00 | 50.00 | NA | 0.00 | 0.00 | 50.00 | 50.00 | 50.00 | 100.00 | NA | 50.00 | NA | 0.00 | 0.00 | 50.00 |
| *Klebsiella* sp. | 7 | 0.00 | 100.00 | 100.00 | 33.33 | 100.00 | 50.00 | 80.00 | 50.00 | 33.33 | 66.67 | 33.33 | NA | 50.00 | 33.33 | 33.33 | 33.33 | 33.33 | 50.00 | 50.00 | 40.00 | NA | 0.00 | 50.00 | 16.67 |
